# Influence of PLLA/PCL/HA Scaffold Fiber Orientation on Mechanical Properties and Osteoblast Behavior

**DOI:** 10.3390/ma12233879

**Published:** 2019-11-24

**Authors:** Lilian de Siqueira, Nilza Ribeiro, Maria B. A. Paredes, Liliana Grenho, Cassilda Cunha-Reis, Eliandra S. Trichês, Maria H. Fernandes, Susana R. Sousa, Fernando J. Monteiro

**Affiliations:** 1 Laboratório de Biocerâmicas, Instituto de Ciência e Tecnologia, UNIFESP, 12231-280 São José dos Campos (SP), Brazileliandra.sousa@unifesp.br (E.S.T.); 2 i3S—Instituto de Investigação e Inovação em Saúde, Universidade do Porto, 4200-135 Porto, Portugal; ribeironilza@gmail.com (N.R.); beleniarteaga11@gmail.com (M.B.A.P.); fjmont@fe.up.pt (F.J.M.); 3 INEB—Instituto de Engenharia Biomédica, Universidade do Porto, 4200-135 Porto, Portugal; 4 Departamento de Engenharia Metalúrgica e de Materiais, Faculdade de Engenharia, Universidade do Porto, 4200-465 Porto, Portugal; 5 Laboratory for Bone Metabolism and Regeneration, Faculdade de Medicina Dentária, FMDUP, 4200-393 Porto, Portugal; liliana.grenho@gmail.com (L.G.); mhfernandes@fmd.up.pt (M.H.F.); 6 LAQV/REQUIMTE—Universidade do Porto, 4051-401 Porto, Portugal; 7 Laboratório Associado, Escola Superior de Biotecnologia, CBQF—Centro de Biotecnologia e Química Fina, Universidade Católica Portuguesa, 4169-005 Porto, Portugal; cassilda_reis@hotmail.com; 8 ISEP—Instituto Superior de Engenharia do Porto, Instituto Politécnico do Porto, 4200-072 Porto, Portugal

**Keywords:** bone-like biomaterial, PLLA/PCL/HA scaffolds, nanohydroxyapatite, electrospinning/electrospraying technique, random and aligned fibers, mechanical properties, osteoblasts

## Abstract

Scaffolds based on aligned and non-aligned poly (L-lactic acid) (PLLA)/polycaprolactone (PCL) fibers obtained by electrospinning, associated to electrosprayed hydroxyapatite (HA) for tissue engineering applications were developed and their performance was compared in terms of their morphology and biological and mechanical behaviors. The morphological results assessed by scanning electron microscopy showed a mesh of PLLA/PCL fibers (random and perfectly aligned) associated with aggregates of nanophased HA. Fourier transform infrared spectrometry confirmed the homogeneity in the blends and the presence of nanoHA in the scaffold. As a result of fiber alignment a 15-fold increase in Young’s Modulus and an 8-fold increase in tensile strength were observed when compared to non-aligned fibers. In PLLA/PCL/HA scaffolds, the introduction of nanoHA caused a remarkable improvement of the mechanical strength of this material acting as a reinforcement, enhancing the response of these constructs to tensile stress. In vitro testing was evaluated using osteoblast (MC3T3-E1) cells. The results showed that both fibrous scaffolds were able to support osteoblast cell adhesion and proliferation and that fiber alignment induced increased cellular metabolic activity. In addition, the adhesion and proliferation of *Staphylococcus aureus* were evaluated and a lower number of colony forming units (CFUs) was obtained in the scaffolds with aligned fibers.

## 1. Introduction

The use of biological substitutes (usually scaffolds) for the functional restoration of organs and tissues damaged as a result of disease or trauma is one of the objectives of the tissue engineering approach in regenerative medicine. In this context, polymeric scaffolds have been studied as substitutes for bone tissue regeneration [1].

Among the polymers used in the production of the scaffolds, poly(l-lactic acid) (PLLA) and polycaprolactone (PCL) have emerged prominently and are among the most promising and widely studied alternatives [2,3] because they display numerous desirable characteristics to be used as implants, i.e., they are biocompatible, bioresorbable and biodegradable and they produce promising results in clinical use [4]. However, their low bioactivity, hydrophobic behavior and long term degradation in vivo may limit their application [5]. The inclusion of calcium phosphate particles inside the polymer matrix or as a coating on its surface may be a solution to address these limitations [6]. Hydroxyapatite (HA), is one of the most widely used biomaterials clinically in bone tissue engineering, due to its biocompatibility and osteoconductivity [7], therefore it may be a good optional coating for the polymer matrix.

Recently, the use of electrospun fibers in biomedical applications has attracted considerable attention due to their capacity to mimic the hierarchical structure of the extracellular bone matrix [8]. An innovative approach has been exploited to obtain polymer fibers partially coated with nanoparticles aggregates, resulting from simultaneous electrospinning of a polymeric solution and electrospraying of a nanoparticle aggregate dispersion. The joint use of these electrodynamic techniques leads to increased nanoparticle exposure, increasing the interactions of both chemistries with the local environment and therefore the surface-dependence characteristics of the nanocomposite fibers, particularly their antimicrobial properties [9,10,11,12,13,14].

Fibers of biodegradable polymers coated with bioactive nanoparticles have been successfully obtained by electrospinning/electrospraying techniques as shown in the literature [15,16]. Such studies showed that by combining gelatin electrospinning and HA electrospraying resulted in nanofibrous scaffolds performing better in terms of cell proliferation, alkaline phosphatase (ALP) activity and mineralization, making them potential substrates for bone tissue regeneration [15]. In another study, Jahangiri prepared nanofibers and nanobeads of triamcinolone acetonide with anti-inflammatory properties. PLGA nanoformulations loaded with triamcinolone acetonide were prepared by an electrospraying method. The results showed that besides displaying controlled-releasing properties, those samples induced drug amorphization during the electrospinning process. They also presented high surface areas and porous structures, thus improving the dissolution profile of poorly water-soluble drugs and providing more efficient drug delivery [16]. In a previous work, Ribeiro et al. [17] designed a material that mimics the extracellular matrix of bone, where nanoHA agglomerated onto electrospun type I collagen nanofibers increased the adhesion and metabolic activity of osteoblasts. This new collagen-nanoHA composite may be used in healing bone defects or as a membrane for tissue regeneration and in treating bone diseases.

In this framework, the goal of the present study was to obtain aligned and non-aligned fibrous scaffolds, using simultaneous electrospinning (of PLLA/PCL) and electrospraying (of nanoHA) and to investigate how fiber orientation and nanoHA coating could affect osteoblast adherence and proliferation to promote bone formation. In addition, microbiological tests regarding the adherence and proliferation of *Staphylococcus aureus* on PLLA/PCL/HA scaffolds were performed.

## 2. Materials and Methods

### 2.1. Materials

The polymers used in this work were PLLA (mol wt 85,000–160,000) and PCL (mol wt 70,000–90,000), both supplied by Sigma-Aldrich (St. Louis, MO, USA). The solvents used were chloroform [CHCl_3_, 99%], supplied by Merck (Darmstadt, Germany) and methanol [CH_3_OH, 99%], supplied by Sigma-Aldrich. Nanohydroxyapatite (nanoHA), named nanoXIM – Hap102 (hydroxyapatite aqueous paste 15% wt) was supplied by Fluidinova S.A. (Moreira da Maia, Portugal).

### 2.2. Electrospinning and Electrospraying

PLLA/PCL blend solutions were obtained by dissolving PLLA and PCL in chloroform. Once the dissolution was complete, methanol was added until a 75/25 v/v solution was obtained, that was magnetically stirred for 12 h at room temperature, to obtain a final concentration of 1.2 g/mL. A syringe (5 mL, using a 21 G needle,) was loaded with the solution which was electrospun at 1 mL/h under a 18 kV electrostatic field onto a rotating collector, at two rotation speeds: 400 rpm (non-aligned fibers) and 3800 rpm (aligned fibers), and at 120 mm from the needle tip. Electrospraying of nanoHA 3.5% (v/v) (particle size of 126 nm ± 2 nm) was carried out simultaneouslly. A syringe (10 mL, using a 21 G needle) was loaded with nanoHA solution to be electrosprayed at 2 mL/h and 18 kV onto the PLLA/PCL fibers at a distance of 120 mm from the collector [17]. Electrospinning and electrospraying processes took place simultaneously for 75 min at 25 °C and 31% to 54% relative humidity.

### 2.3. Physical and Mechanical Characterization of Fibers

Fibers morphology was characterized by scanning electron microscopy (SEM) using a Quanta 400 FEG E SEM/EDAX Pegasus X4M system (FEI, Hillsboro, OR, USA; high vacuum/10.0 kV). The diameters of thirty randomly chosen fibers was taken from SEM images of three different samples using the ImageJ 6.0 software (version 1.49). The mean diameter of the fibers and its distribution were obtained from a Gauss curve. 

The chemical composition was obtained through Fourier transform infrared spectroscopy (FT-IR), using a Frontier FT-IR spectrometer (Perkin-Elmer, Waltham, MA, USA) with a scanning range of 400 to 4000 cm^−1^. For analysis, 0.2 g of PLLA/PCL fibers or PLLA/PCL/HA scaffolds were ground and KBr pellets were prepared to achieve a spectral resolution of 4 cm^−1^. One hundred scans were accumulated per sample.

The mechanical properties of the fiber mats were assessed based on ASTMD882-02 methods, on wet samples, by uniaxial tensile testing using a texture analyzer (TA. XT. Plus^®^, Stable Micro Systems, Surrey, UK). Mats were cut into rectangular strips of 0.75 cm × 3 cm and maintained immersed in phosphate-buffer saline (PBS), at room temperature, for at least 4 h prior to testing, in order to achieve the hydration equilibrium. Then, the thickness of 6 samples of each experimental condition was measured with a digital micrometer (MI20, Adamel Lhomargy, Ivry sur Seine Cedex, France). To avoid jaw slippage and jaw breaks, the edges of the samples were mounted between two strips of thin card and held together using double sided adhesive tape. Then, the samples were mounted on the grips and tests were performed at a cross-head speed of 5 mm/min. Samples hydration during preparation and testing was maintained by regular PBS spraying. Young’s Modulus, tensile strength and elongation at break were calculated from the obtained stress/strain curves. The results represent the average of six samples.

### 2.4. Cell Culture

MC3T3-E1 cells (ATCC^®^ CRL-2593), established as an osteoblastic cell line from normal mouse calvaria, were grown in alpha minimum essential medium (α-MEM, Gibco, Carlsbad, CA, USA) supplemented with 10 % (v/v) fetal bovine serum (FBS) (Gibco) and 100 U/mL penicillin- 100 µg/mL streptomycin (Gibco). Cells were cultured in 25 cm^2^ plastic culture flasks (Corning, Nova York, NY, USA) and incubated in a humidified incubator at 37 °C and 5 % CO_2_. Freshly confluent MC3T3-E1 cells were rinsed with PBS, followed by incubation in trypsin/EDTA (0.25 % trypsin, 1 mM EDTA; Sigma-Aldrich) for 5 min at 37 °C and re-suspended in supplemented medium. 

#### 2.4.1. Cell Seeding on Biomaterials Surfaces

Scaffolds were sterilized by immersion in sequential 90%, 70% and 50% (v/v) ethanol/water solutions for 10 min and further incubated with α-MEM for 30 min. Cells were seeded on the 4 different substrates at a cell density of 2 × 10^4^ cells/mL (electrospun PLLA/PCL fibers aligned and non-aligned, with and without electrosprayed HA). Cells culture times were 1, 4, 7 and 14 days. The medium was changed every two days. For each material and culture period, six samples without cells were incubated with complete medium in the same way and used as blanks. MC3T3-E1 cells cultured on tissue culture polystyrene (TCPS) were used as control. 

#### 2.4.2. Cell Metabolic Activity

After 1, 4, 7 and 14 days of cell culture the metabolic activity of MC3T3-E1 cells was evaluated using a resazurin-based assay [18]. Briefly, 10% (v/v) resazurin solution (Sigma-Aldrich) at 0.1 mg/mL was added onto each well. After 3 h at 37 °C, fluorescence was measured using λ_ex_ = 530 nm and λ_em_ = 590 nm in a microplate reader (Synergy MX, BioTek^®^ Instruments, Inc., Winooski, VT, USA). The fluorescence value corresponding to the non-seeded substrates was subtracted. Results correspond to the mean ± standard deviation of six cultured samples.

#### 2.4.3. Cell Distribution and Cell Morphology 

The distribution and morphology of MC3T3-E1 cells on 3D scaffolds was studied by laser scanning confocal microscopy (LSCM) and SEM. For immunostaining of F-actin cytoskeleton and nuclei, cell-seeded surfaces were fixed with 4% para-formaldehyde for 15 min, permeabilized with 0.1% Triton X-100 for 5 min and incubated in 1% BSA for 30 min at room temperature (RT). Cell cytoskeleton filamentous actin was visualized using Alexa Fluor^®^ 594 Phalloidin (1:200 in BSA 1%, Molecular Probes^®^, Karlsruhe, Germany) for 20 min under darkness. Subsequently, cells were PBS washed and 4′, 6-diamidino-2-phenylindole (Vectashield/DAPI, Sigma-Aldrich) dye was used for 10 min under darkness, to counterstain cell nuclei. The images were acquired on a Zeiss LSM 510 confocal microscope (Leica Microsystems, Wetzlar, Germany) using a Plan-Apochromat 40× oil objective. Images were processed and quantified using Leica application Suite X version 3.3.0.16799 software. For the SEM observations, cell-seeded samples fixed with 1.5% glutaraldehyde were dehydrated with an increasing ethanol–water gradient and dried using hexamethyldisilazane (Sigma-Aldrich). The SEM studies were carried out using a high-resolution scanning electron microscope with X-ray microanalysis system (JSM 6301F/Oxford INCA Energy 350, JEOL, Peabody, MA, USA). Samples were sputtered with a thin film of Pd-Au, using the SPI Module Sputter Coater. The conditions in which images and spectrum were obtained are indicated in the respective labels.

### 2.5. Bacterial Culture

The total number of colony-forming units (CFU) per mL of *Staphylococcus aureus* (ATCC 25923) bacteria adhesion on scaffolds surfaces was followed. Bacteria were grown on tryptic soya broth (TSB, Merck). Nutrient agar (NA, Liofilchem, Roseto degli Abruzzi TE, Italy) plate inoculated with respective bacteria S. aureus strain was incubated at 37 °C for 18 h. Bacterial suspensions were adjusted by measuring optical density (600 nm) using a Lambda 35 UV/Vis spectrometer (Perkin Elmer).

#### 2.5.1. Bacterial Adhesion on Biomaterials Surfaces

Materials were UV sterilized for 15 min and washed with 0.9 % NaCl. Samples were transferred to 96-well, clear flat bottom polystyrene not treated microplates (Corning). Two hundred µL of bacterial suspension of 1 × 10^8^ CFU/mL or 200 µL of TBS were placed on each corresponding sample and incubate at 37 °C for 2 h to promote bacterial adhesion and growth onto the scaffolds surfaces.

#### 2.5.2. Colony-Forming Units (CFU)

After incubation period, substrates were washed with 0.9% NaCl to remove non-adherent bacteria, transferred to tubes containing 1 mL of 0.9% NaCl and sonicated for 10 min in an ultrasound bath (Sonorex Digitec Bath 35 kHz, Bandelin, Berlin, Germany) to release all adherent bacteria. The sonicated solutions were used to make serial dilutions, and these were placed onto NA culture plates and incubated at 37 °C for 18 h. Three replicates for each condition were used. Afterwards, the number of adherent bacteria was counted, and the number of CFU/mL was determined. TCPS samples were used as control.

### 2.6. Statistical Analysis

Data analysis was performed using Graph Pad Prism software, version 5.00.288 for Windows. Statistical analysis specifically for cell metabolic activity data was assessed using nonparametric one-way ANOVA and all values are expressed as mean ± sd. Bonferroni correction was used in all statistical tests with multiple groups. For the microbial activity assays the nonparametric Kruskal–Wallis test was applied to perform statistical comparisons between groups, and P values of less than 0.05 were considered statistically significant.

## 3. Results

**Figure 1:**
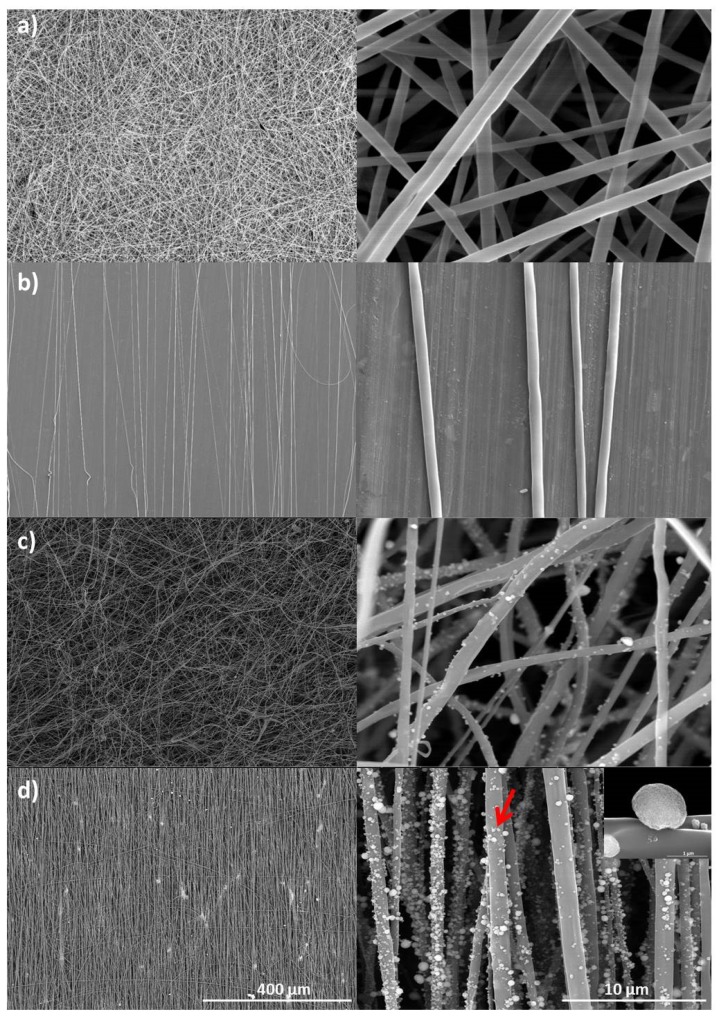
SEM images of electrospun and electrosprayed fibers: (**a**) non-aligned PLLA/PCL; (**b**) aligned PLLA/PCL; (**c**) non-aligned PLLA/PCL/HA; (**d**) aligned PLLA/PCL/HA, with different magnifications. Red arrow indicates the aggregates of nanophased HA deposited onto the fiber surface.

The fibers of PLLA/PCL obtained by electrospinning were smooth, homogeneous, and without defects, as observed in the SEM micrographs (Figure 1a,b). In particular, when the collector speed was 400 rpm, a random mesh interconnected fibrous network without beads was obtained (Figure 1a). 

**Figure 2:**
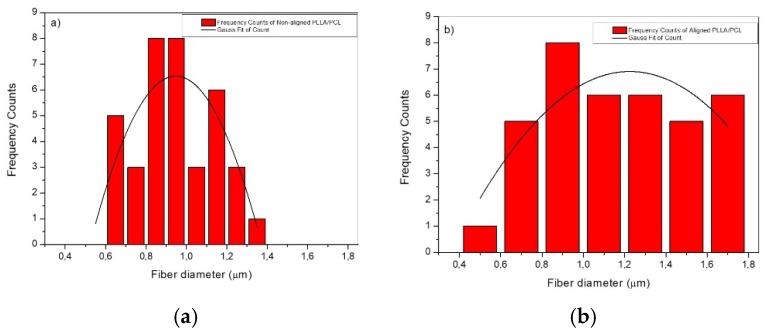
Fiber diameter distribution in (**a**) non-aligned PLLA/PCL; (**b**) aligned PLLA/PCL.

On the other hand, when he collector speed was increased to 3800 rpm, the fibers tended to be aligned (Figure 1b). According to Liao et al. [19] this rotation may be considered as an alignment speed, matching the one of evaporated jet depositions, the fibers being rapidly taken up tightly on the collector surface circumferentially, resulting in high alignment. The average diameters of the PLLA/PCL aligned, and non-aligned fibers were obtained from the fiber distribution data shown in Figure 2. Corresponding average fiber diameters of aligned and non-aligned PLLA/PCL are 0.94 ± 0.04 μm (with diameter distribution between 0.65 and 1.24 μm) and 1.22 ± 0.12 µm (with diameter distribution between 0.61 and 1.75 μm), respectively. In addition, the electrospraying process provided good homogeneity in terms of nanoHA distribution on polymer fibers surfaces (Figure 1c,d).

**Figure 3:**
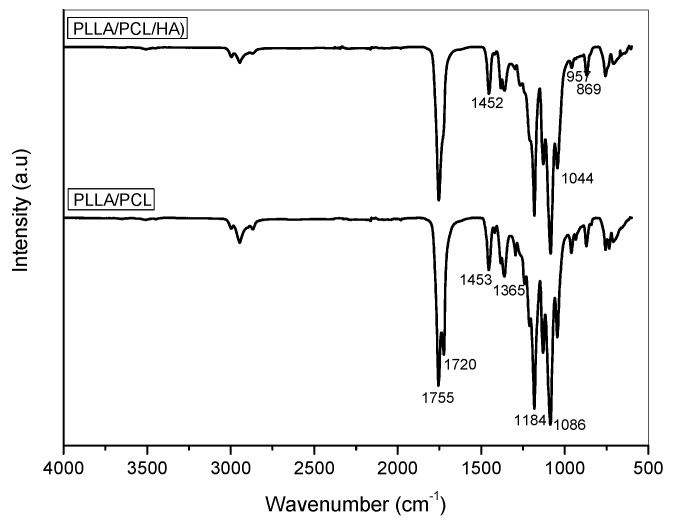
FT-IR spectra of electrospun PLLA/PCL fibers and PLLA/PCL/HA scaffolds.

The FT-IR results of PLLA/PCL fibers and PLLA/PCL/HA scaffolds are shown in Figure 3. The corresponding peaks of PCL/PLLA fibers represent related peaks of both the polymers with respect to the proportion of each polymer, justifying blend homogeneity. The presence of nanoHA particles was also confirmed by their characteristic absorption bands. Concerning the micro-structure of PLLA fibers shown in Figure 2 the absorbance observed at 1755 cm^−1^ is due to the carbonyl stretching (C=O) and at 1453 cm^−1^ to asymmetric vibration of the CH_3_ lateral groups [20]. The absorbances at 1184 and 1086 cm^−1^ correspond to stretching vibrations of the C–O–C groups. For PCL, the main absorption band occurs at 1720 cm^−1^, corresponding to the carbonyl group [21]; the band at 1365 cm^−1^ correspond to folds of the CH_3_ group. These groups indicate the presence of both polymers (PLLA/PCL) in the fibers. In the FT-IR spectrum of the PLLA/PCL/HA scaffolds besides the polymer’s characteristic bands, the characteristic bands of nanophased HA:PO_4_^2−^ bands (υ_3_ ~ 1044 and υ_1_ ~ 957 cm^−1^), were revealed. Characteristic bands of the carbonate group could also be observed, namely those corresponding to the υ_3_ vibration of C–O (1452 cm^−1^) and the υ_2_ vibrations (863 cm^−1^) [22].

**Figure 4:**
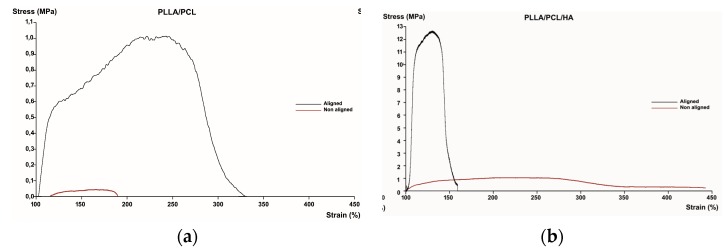
Representative stress/strain plots of electrospun PLLA/PCL fibers (**a**) and PLLA/PCL/HA scaffolds (**b**).

The mechanical performance of hydrated PLLA/PCL fibers and PLLA/PCL/HA scaffolds was evaluated using uniaxial tensile tests. Figure 4 shows representative stress/strain plots evidencing a strong influence of both composition and alignment on tensile strength.

Randomly oriented PLLA/PCL fibers showed poor mechanical performance, with low Young’s Modulus and tensile strength. However, aligning the fibers led to a 15-fold increase in stiffness (Young’s Modulus) and an 8-fold increase in tensile strength, as observed in Table 1.

**Figure 5:**
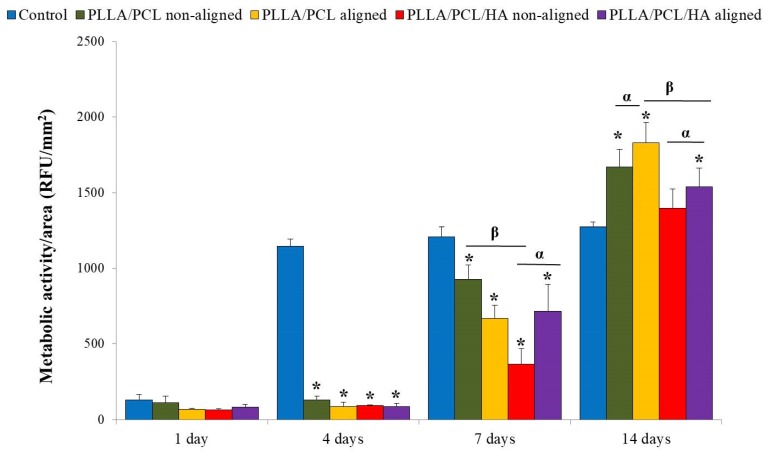
Metabolic activity of MC3T3-E1 cells cultured on the electrospun non-aligned PLLA/PCL fibers, electrospun aligned PLLA/PCL fibers, non-aligned PLLA/PCL/HA scaffolds and aligned PLLA/PCL/HA scaffolds. The results are expressed in terms of relative fluorescence units (RFU) per unit area (mm^2^). MC3T3-E1 cells cultured on TCPS were used as control. Values are the average ± SD of six cultures. * indicates a statistically significant difference from the control cultures (TCPS). α indicates a statistically significant difference from oriented and non-oriented substrates. β indicates a statistically significant difference from substrates with and without HA (*p* ≤ 0.05).

Regarding the biological tests, the cellular metabolic activity of MC3T3-E1 cells increased with the culture time for all scaffolds, indicating that the four groups were non-cytotoxic and able to support osteoblast cells adhesion and growth (Figure 5) and after 14 days of culture, MC3T3-E1 cells showed higher metabolic activity than control. Interestingly enough, significantly higher cellular metabolic activity of MC3T3-E1 cells growing on the aligned matrices was observed as compared to non-aligned ones, especially for the longest culture time point (Figure 4). The presence of hydroxyapatite did not induce any major differences in the metabolic activity of these cells cultured.

**Figure 6:**
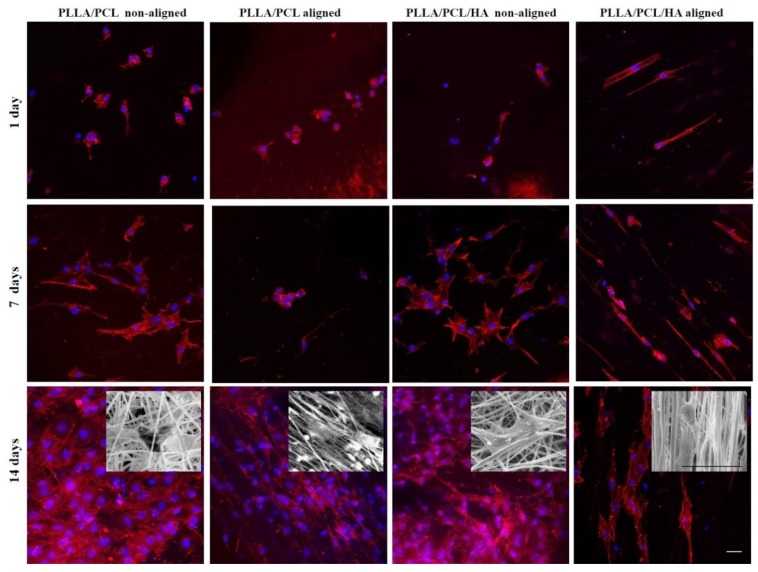
Morphology and cytoskeletal organization followed by confocal imaging of MC3T3-E1 cells cultured on the electrospun non-aligned PLLA/PCL fibers, electrospun aligned PLLA/PCL fibers, non-aligned PLLA/PCL/HA scaffolds and aligned PLLA/PCL/HA scaffolds. F-actin was indicated in red while the cells’ nuclei were counterstained with DAPI (blue). SEM images of MC3T3-E1 cells cultured on the matrices tested at day 14. Scale bars: 30 μm.

Regarding the morphology of MC3T3-E1 at day 1, cells were attached and spread-out across the surface, with characteristic spherical or spindle-shaped appearance (Figure 6). Cell distribution was completely different depending on the biomaterial’s network architecture. Particularly, osteoblasts adopted a rounder morphology with less extended cytoskeleton on non-oriented matrices and a random distribution on their surfaces. While, for the oriented structures the MC3T3-E1 were spread-out with spindle-like morphology and alignment according to the orientation of the matrices themselves (Figure 6). As expected, all the surfaces presented better cell coverage at day 14 when compared to the earlier time points. SEM images showed that MC3T3-E1 cells cultured on the different scaffolds completely adhered to their surfaces, reaching a fusion-like state, making it difficult to distinguish cells from the substrate material (meshes of PLLA/PCL fibers and HA agglomerates).

**Figure 7:**
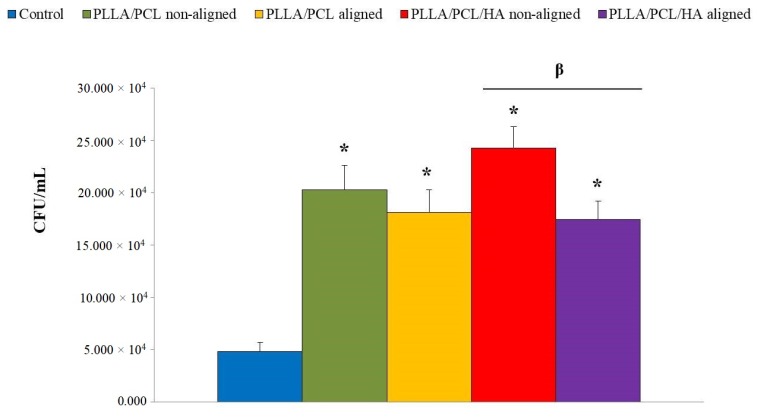
The total number of colony-forming units (CFU) per mL of *Staphylococcus aureus* adhesion on the electrospun non-aligned PLLA/PCL fibers, electrospun aligned PLLA/PCL fibers, non-aligned PLLA/PCL/HA scaffolds and aligned PLLA/PCL/HA scaffolds. Bacterial adhesion on TCPS was used as control. * indicates a statistically significant difference from control cultures. β indicates a statistically significant difference with respect to substrates with and without HA (*p* ≤ 0.05).

Regarding the number of colony-forming units (CFU)/mL formed, there was no statistically significant difference in *Staphylococcus aureus* adhesion between matrices with and without HA on their composition (Figure 7). Yet, our findings highlight a distinct number of bacteria depending on the fibers orientation that compose the different membranes. In fact, the aligned fibers revealed a lower number of CFU when compared to non-aligned fibers samples, being statistically significant for the PLLA/PCL/HA matrices (Figure 7).

## 4. Discussion

The rotation speed of the collector cylinder significantly impacted the quality of the alignment of the obtained fibers, as it may be observed in the micrographs of the fibers (Figure 1) being in agreement with the literature, as reported by Inai et al. [23] and Mathews et al. [24]. Such evidence corroborates the explanation for fiber alignment when using the rotating cylinder strategy, where fibers are drawn into the cylinder at the same time as they are stretched due to the cylinder rotation. Thus, when rotation and collection speeds are tuned, a higher fiber alignment efficiency occurs. If, on the other hand, the speed of rotation is well above the collecting speed, the fibers are broken and the obtained mesh will have discontinuous and brittle fibers. Finally, if the speed of rotation is too low, the collector will behave as a fixed collector, having no aligning effect. Under the experimental conditions tested, the results suggest that cell adhesion signaling can be triggered by potential intrinsic effects exerted by the fiber architecture [8].

If strong fibers with high elastic modulus are desired, then molecular polymeric chains should be fully stretched and aligned along the fiber axis and with only a very small number of chain ends or defects present. The stretched fibers provide high stiffness while the chain ends provide high tenacity [25].

This principle also applies to the production highly performing electrospun fibers. Obtaining molecular orientation along chain extension will lead to the production of fiber mats with improved stiffness and strength, hence, numerous methods that improve fiber alignment, such as using collectors with specific designs (rotating, parallel, water bath) or applying extra auxiliary forces (magnetic, electric, centrifugal) have been developed [26]. Indeed, several studies have shown that fiber orientation plays a major role on the mechanical performance of fiber mats. For example, when evaluating the influence of fiber orientation on the mechanical properties of poly(vinylidene fluoride), Maciel et al. [27] have found that the Young’s modulus depended significantly on the angle between the stretching direction and the fiber direction, with significantly higher values being obtained for 0° angle (10-fold increase). When the fibers were randomly oriented, however, the young´s Modulus was independent from the deformation direction. They have also observed that, with the applied stress, all samples underwent a reorientation of the fibers towards the stretching direction and a decrease in fiber diameter. Mubyana et al. [28] have reported a similar trend when studying the effect of alignment on the mechanical performance of PCL nanofiber mats. However, these authors have further reported that the mat thickness also played an important role. They observed that the value of failure strain was similar for different mat thicknesses with similar elongation values, independently of the thickness. However, similar behaviors were observed both for the aligned fibers and randomly oriented fibers groups, in terms of decreasing toughness, ultimate tensile strength and short-range modulus with increasing mat thickness. There were thickness differences within the several experimental groups of the present study. Nevertheless, since the magnitude of the alignment effect was so great, it is most likely that, if existing, the contribution of thickness to the overall results would have been negligible.

The same trend was observed for PLLA/PCL/HA scaffolds—fiber alignment across the stretching direction led to increased stiffness and tensile strength. In this case, introducing nanoHA has caused a remarkable improvement on the mechanical properties of these blends. Along with conferring bioactive properties to the scaffolds, nanoHA also acted as a reinforcement, enhancing the response of these constructs to tensile stress. Jose et al. [29] have also reported similar results, using PLGA nanofibers reinforced with nanoHA.

Regarding the elongation at break, opposite behaviors were observed for PLLA/PCL fibers and PLLA/PCL/HA scaffolds. In the first case, fiber alignment led to an increased elongation before failure. In fact, this finding was the opposite of what was expected. When under stress, fibers usually rotate to get aligned with the load direction and attempt to resist to deformation. When fiber mats are randomly oriented, greater rotation and reorientation are required before alignment with the load axis is achieved and hence they are more extensible. Aligned fiber mats require minimal rotation to get alignment with de loading direction and therefore fail faster [28], as it was observed in the PLLA/PCL/HA scaffolds. Wong et al. [30] have reported that fiber diameter plays a crucial role on structural and mechanical characteristics of PCL nanofibers. Those authors found that reducing fiber diameter improved crystallinity and molecular orientation, resulting in less compliant samples. Fiber diameter in aligned PLLA/PCL mats was thicker than in randomly oriented samples (Figure 2). It is possible that the contribution of fiber diameter to the extensibility of these samples might have been more relevant than that of the fiber alignment, leading to higher elongation at break values for the aligned samples.

The effect of the biomaterial’s network architecture as well as the presence of hydroxyapatite on MC3T3-E1 metabolic activity, distribution and morphology, was followed after 1, 4, 7 and 14 days of cell culture, as presented in Figure 5 and Figure 6. Regarding cellular interactions, all the electrospun fiber construct were cytocompatible, withstanding long-term MC3T3-E1 osteoblast adhesion and growth. There were no significant differences in terms of MC3T3-E1 cells’ metabolic activity and morphology deriving from the addition of nanophased HA in the successfully developed matrices, as opposed to what was reported by other authors [17,22,31,32]. The difference in size between the electrosprayed nanoHA agglomerates and the PLLA/PCL micro fibers, might explain the absence of effect on cells behavior. Therefore, in the cell/material interface the micro-network topology will prevail in detriment of the presence of the hydroxyapatite nanocrystals that line the micro fibers. On the contrary, the topography of biomaterial surfaces appeared to contribute to the significant growth and increased metabolic activity of MC3T3-E1 cells. Importantly enough, these cells adopted a specific shape and distribution, following the orientation of the constructs network. Regarding the microbiological tests, the adherence and proliferation of *Staphylococcus aureus* on PLLA/PCL/HA scaffolds was observed as expected, since the adherence of this bacterium strain to nanoHA was previously reported [33]. However, it was surprising to note that the number of colony-forming units (CFU) on aligned scaffolds surfaces was significantly lower than on non-aligned ones (Figure 7), which is in accordance with cell culture results (Figure 5).

## 5. Conclusions

Aligned and non-aligned PLLA/PCL fibers and PLLA/PCL/HA scaffolds were successfully produced by a simultaneous electrospinning-electrospraying process. The fibers showed a porous, interconnected and fibrous structure with diameters ranging between 0.61–1.75 μm. Alignment of the fibers led to a 15-fold increase in stiffness (Young’s Modulus) and an 8-fold increase in tensile strength, compared to the results obtained for non-aligned fibers. The same trend was observed for PLLA/PCL/HA scaffolds. In this case, the introduction of aggregates of nanophased HA caused a remarkable improvement of the mechanical properties of these blends, acting as a reinforcement, enhancing the response of these constructs to tensile stress. The tensile strength of cortical bone is about 2.0–12.0 MPa. Hence, the tensile strength of the present aligned PLLA/PCL/HA scaffolds falls in this range. The best scaffold obtained, aiming at future applications in tissue engineering, was the aligned PLLA/PCL/HA, because it showed significantly higher cellular metabolic activity and lower CFU numbers when compared to non-aligned fibers. The presence of hydroxyapatite did not induce any major differences in the biological studies. These aligned synthetic biomimetic ECM’s may be promising candidates for bone tissue applications, as functional membranes to guide the growth, capable to both enhancing cell response when applied to fill small bone defects and when coating the bone bonding surfaces of load bearing implants or prostheses.

## Figures and Tables

**Table 1:** Mechanical behavior of electrospun PLLA/PCL fibers and PLLA/PCL/HA scaffolds.

Samples	Thickness (µm)	Young’s Modulus (MPa)	Tensile Strength (MPa)	Elongation at Break (%)
**PLLA/PCL non-aligned**	65.2 ± 12.1	0.002 ± 0.001	0.12 ± 0.01	174.15 ± 9.54
**PLLA/PCL aligned**	45.8 ± 7.2	0.03 ± 0.01	0.99 ± 0.09	252.74 ± 15.53
**PLLA/PCL/HA non-aligned**	30 ± 8	0.1 ± 0.02	1.66 ± 0.27	329.54 ± 14.12
**PLLA/PCL/HA aligned**	16 ± 4	2.99 ± 0.63	11.32 ± 1.94	131.83 ± 6.82

## References

[1] Zong H., Xia X., Liang Y., Dai S., Alsaedi A., Hayat T., Kong F., Pan J.H. (2017). Designing function-oriented artificial nanomaterials and membranes via electrospinning and electrospraying techniques. Mater. Sci. Eng. C..

[2] Yang F., Murugan R., Wang S., Ramakrishna S. (2005). Electrospinning of nano/micro scale poly (L-lactic acid) aligned fibers and their potential in neural tissue engineering. Biomaterials.

[3] Lee K.H., Kim H.Y., Khil M.S., Ra Y.M., Lee D.R. (2003). Characterization of Nano-Structured Poly(ɛ-Caprolactone) Nonwoven Mats via Electrospinning. Polymer.

[4] Griffith L.G. (2000). Polymeric biomaterials. Acta Mater..

[5] Ma P.X. (2004). Scaffolds for tissue fabrication. Mater. Today.

[6] Zhou H., Lawrence J.G., Bhaduri S.B. (2012). Fabrication aspects of PLA-CaP/PLGA-CaP composites for orthopedic applications. A review. Acta Biomater..

[7] Liao G.Y., Jiang S., Xia H., Jiang K. (2012). Preparation and characterization of aligned PLLA/PCL/HA composite fibrous membranes. J. Macromol. Sci. A.

[8] Cristofaro F., Gigli M., Bloise N., Honglin C., Bruni G., Munari A., Moroni L., Lotti N., Visai L. (2018). Influence of nanofiber chemistry and orientation of biodegradable poly(butylene succinate)-based scaffolds on osteoblast differentiation for bone tissue regeneration. Nanoscale.

[9] Jaworek A., Krupa A., Lackowski M., Sobczuk A.T., Czech T., Ramakrishna S., Sundarrajan S., Pliszka D. (2009). Nanocomposite fabric formation by electrospinning and electrospraying technologies. J. Electrost..

[10] Bock N., Woodruff M.A., Hutmacher D.W., Dargaville T.R. (2011). Electrospraying, a Reproducible Method for Production of Polymeric Microspheres for Biomedical Applications. Polymers.

[11] Vitchuli N., Shi Q., Nowak J., Kay K., Caldwell J.M., Breidt F., Bourham M., McCord M., Zhang X. (2011). Multifunctional ZnO/Nylon 6 nanofiber mats by an electrospinning-electrospraying hybrid process for use in protective applications. Sci. Technol. Adv. Mater..

[12] Ramier J., Bouderlique T., Stoilova O., Manolova N., Rashkov I., Langlois V., Renard E., Albanese P., Grande D. (2014). Biocomposite scaffolds based on electrospun poly (3-hydroxybutyrate) nanofibers and electrosprayed hydroxyapatite nanoparticles for bone tissue engineering applications. Mater. Sci. Eng. C..

[13] Virovska D., Paneva D., Manolova N., Rashkov I., Karashanova D. (2014). Electrospinning/electrospraying vs. electrospinning: A comparative study on the design of poly (l-lactide)/zinc oxide non-woven textile. Appl. Surf. Sci..

[14] Rodríguez-Tobías H., Morales G., Ledezma A., Romero J., Grande D. (2014). Novel antibacterial electrospun mats based on poly (d,l-lactide) nanofibers and zinc oxide nanoparticles. J. Mater. Sci..

[15] Francis L., Venugopal J., Prabhakaran M.P., Thavasi V., Marsano E., Ramakrishna S. (2010). Simultaneous electrospin–electrosprayed biocomposite nanofibrous scaffolds for bone tissue regeneration. Acta Biomater..

[16] Jahangiri A., Davaran S., Fayyazi B., Tanhaei A., Payab S., Adibkia K. (2014). Application of electrospraying as a one-step method for the fabrication of triamcinolone acetonide-PLGA nanofibers and nanobeads. Colloids Surf. B.

[17] Ribeiro N., Sousa S.R., van Blitterswijk C.A., Moroni L., Monteiro F.J. (2014). A biocomposite of collagen nanofibers and nanohydroxyapatite for bone regeneration. Biofabrication.

[18] O’Brien J., Wilson I., Orton T., Pognan F. (2000). Investigation of the Alamar Blue (resazurin) fluorescent dye for the assessment of mammalian cell cytotoxicity. Eur. J. Biochem..

[19] Liao G., Jiang S., Xu X., Ke Y. (2012). Electrospun aligned PLLA/PCL/HA composite fibrous membranes and their in vitro degradation behaviors. Mater. Lett..

[20] Zhang J., Duan Y., Sato H., Tsuji H., Noda I., Yan S., Ozaki Y. (2005). Crystal modifications and thermal behavior of poly(L-lactic acid) revealed by infrared spectroscopy. Macromolecules.

[21] Sasmazel H.T. (2011). Novel hybrid scaffolds for the cultivation of osteoblast cells. Int. J. Biol. Macromol..

[22] Ribeiro C.C., Gibson I., Barbosa M.A. (2006). The uptake of titanium ions by hydroxyapatite particles - structural changes and possible mechanisms. Biomaterials.

[23] Inai R., Kotaki M., Ramakrishna S. (2005). Structure and properties of electrospun PLLA single nanofibres. Nanotechnology.

[24] Matthews J.A., Wnek G.E., Simpson D.G., Bowlin G.L. (2002). Electrospinning of collagen nanofibers. Biomacromolecules.

[25] Yao J., Bastiaansen C., Peijs T. (2014). High strength and high modulus electrospun nanofibers. Fibers.

[26] Yuan H., Zhou Q., Zhang Y., Afshari M. (2017). 6 - Improving fiber alignment during electrospinning. Electrospun Nanofibers.

[27] Maciel M.M., Ribeiro S., Ribeiro C., Francesko A., Maceiras A., Vilas J.L., Lanceros-Méndez S. (2018). Relation between fiber orientation and mechanical properties of nano-engineered poly(vinylidene fluoride) electrospun composite fiber mats. Compos. Part B- Eng..

[28] Mubyana K., Koppes R.A., Lee K.L., Cooper J.A., Corr D.T. (2016). The influence of specimen thickness and alignment on the material and failure properties of electrospun polycaprolactone nanofiber mats. J. Biomed. Mater. Res. A.

[29] Jose M.V., Thomas V., Xu Y., Bellis S., Nyairo E., Dean D. (2010). Aligned bioactive multi-component nanofibrous nanocomposite scaffolds for bone tissue engineering. Macromol Biosci..

[30] Wong S.C., Baji A., Leng S. (2008). Effect of fiber diameter on tensile properties of electrospun poly(ɛ-caprolactone). Polymer.

[31] Song W., Markel D.C., Wang S.X., Shi T., Mao G.Z., Ren W.P. (2012). Electrospun polyvinyl alcohol-collagen-hydroxyapatite nanofibers: a biomimetic extracellular matrix for osteoblastic cells. Nanotechnology.

[32] Hongfei Q.I., Zhihong Y., Hailong R., Nana C., Qingyan Z., Xianglong W., Tingli L. (2016). Bioactivity assessment of PLLA/PCL/HAP electrospun nanofibrous scaffolds for bone tissue engineering. Life Sci..

[33] Barros J., Grenho L., Manuel C.M., Ferreira C., Melo L.F., Nunes O.C., Monteiro F.J., Ferraz M.P. (2014). Influence of nanohydroxyapatite surface properties on Staphylococcus epidermidis biofilm formation. J. Biomater. Appl..

